# Reply to Wolowich and Kwon

**DOI:** 10.1093/cid/ciaa911

**Published:** 2020-07-02

**Authors:** Miao Zhang, Xueting Yao, Cheng Cui, Xu Liu, Haiyan Li, Dongyang Liu

**Affiliations:** 1 Drug Clinical Trial Center, Peking University Third Hospital, Beijing, China; 2 School of Basic Medicine and Clinical Pharmacy, China Pharmaceutical University, Nanjing, China; 3 Department of Cardiology and Institute of Vascular Medicine, Peking University Third Hospital, Beijing, China

To the Editor—We thank Wolowich and Kwon for their letter commenting on our earlier publication [1], and appreciate the opportunity to respond.

The authors stated that “Yao concludes a dosage regimen of oral HCQ [hydroxychloroquine] provides sufficient lung exposure to exceed the viral half-maximal effective concentration (EC_50_) and thus the regimen should be effective in treating COVID-19 patients. This conclusion is not warranted from the data presented,” and further that “these findings … could mislead practitioners to use the drug in COVID-19 [coronavirus disease 2019] patients with little evidence of efficacy.” This significantly misinterpreted our publication. The only conclusion we made was that “HCQ was found to be more potent than CQ [chloroquine] to inhibit SARS-CoV-2 [severe acute respiratory syndrome coronavirus 2] in vitro” [1]. Although one can employ modeling and pharmacology concepts to predict the likelihood of clinical efficacy from in vitro data, given the inherent limitations of any modeling approach and assumptions being made, in vitro efficacy can only be ultimately confirmed through clinical trials. To this end, any modeling analysis has to be fit for purpose. In our article [1], the purpose of physiologically based pharmacokinetic (PBPK) modeling was to provide timely support on dosing decisions for our clinicians, who eventually used safe doses of CQ and HCQ that were approved for other indications to treat patients with COVID-19 in Wuhan, China. Specifically, we tried to use PBPK modeling first to understand why CQ was found to have clinical antiviral effects [2], and then to determine the relative potency between HCQ and CQ based on in vitro EC_50_ values and respective drug models. To date, the clinical antiviral activities of CQ and HCQ remain to be confirmed [3, 4].

Tissue-to-plasma partition coefficient (Kp) is a critical bridging parameter to estimate tissue concentration [5]. As we clearly recognized in our Discussion, we made several assumptions in our analysis that require further validation and refinement. We used a perfusion limited tissue distribution model to mimic the time-dependent lung Kp characteristics [1], where we assumed the same lung Kp characteristics of HCQ as that of CQ (11–547 from 1 hour to 168 hours postdosing) [6], which was similar to lung Kp value in mice (average, 29 × 7.2 from 6 hours to 72 hours postdosing) reported by Chhonker et al [7] and our newly generated monkey data (~200 at 24 hours postdosing; Liu et al, unpublished data). We also benchmarked CQ exposure under 500 mg twice daily for 10 days, which first showed antiviral effect in Chinese patients [2], and calculated a lung tissue to plasma concentration (R_LTEC_) of HCQ under regimen “F” to be greater than CQ. Wolowich and Kwon stated that they used a “slightly modified version of an HCQ model published by Collins et al” [8], and we respectfully disagree. The model by Collins et al was developed in different software with different model assumptions, where we did not see how to simulate lung tissue concentration. As we are not able to see what Wolowich and Kwon simulated, we hereby point out the following assumptions/observations reported in Collins et al’s article [8] that warrant further discussion:

1. Kp was set as a constant rather than a time-dependent function, whereas animal studies [6], as well as the unpublished Liu et al study, suggest time-dependent accumulation of both CQ and HCQ; we tried to capture the time-dependent drug accumulation by using an additional organ in Simcyp, and acknowledged the limitation of this parameterization in our Discussion. We and others are updating these models (Cui et al [9]; Rowland Yeo et al [10]; Zhang et al, unpublished data).
2. The simulated half-life (68–77 hours; see Table 4 of Collins et al [8]) appears to be much shorter than that observed in clinical studies [11, 12] (~40 days) and our article’s simulation results (~20 days) [1].
3. Although Collins et al simulated HCQ concentrations in human lysosomes, these concentrations have not been validated with any nonclinical or clinical data, and HCQ could also be accumulated in other acid cell organs, such as endosome or golgi [13, 14], which could significantly affect lung tissue concentration simulated by Wolowich and Kwon if they just assume HCQ was accumulated in lysosome. Actually, pH increase in acid cell organs, led by HCQ accumulation in these cell organs, was suggested to be a key mechanism to inhibit SARS-CoV-2, although it was not confirmed [15].

We would also like to clarify issues brought up by Wolowich and Kwon, who apparently misunderstood our analysis [1] and the use of Simcyp software:

1. f_a_ is fraction absorbed in intestine, specifically fraction of drug from gut lumen to enterocytes, rather than bioavailability (F). The simulated F, which actually equals f_a _× f_g _× f_h_ (f_g_ and f_h_ being fractions of drug escaping metabolism within enterocytes and liver first-pass metabolism, respectively) was 0.8, and reported F in humans was 0.75 [11, 12].
2. K_a_ of 0.5 hour^−1^ of k_a_ mentioned by Wolowich and Kwon was not found in Tett et al [11], whereas other studies [12, 16] reported 0.194 or 1.15 hour^−1^ in humans, which informed the value of 0.8 hour^−1^ in our article [1].
3. Kp scaler in our PBPK model was not applied to lung tissue because we set lung as an additional organ, which allowed us to mimic the time-dependent tissue distribution profiles (see previous paragraph).
4. We decided not to simulate lysosomal concentration in that we could not validate the lysosome concentration using in vivo data, and again the Simcyp lung model has a different model structure than that reported by Collins et al.

In conclusion, we used PBPK models with the best knowledge available to timely support safe use of CQ and HCQ by our clinicians to treat COVID-19 patients safely in Wuhan and Nanchang City. We declared our assumptions of modeling and acknowledged limitations. Soon after publication, we contributed to medical research by uploading the raw model files to the Simcyp repository so that others can readily apply them [10, 17].

## Notes

***Financial support.*** This work was supported by the Ministry of Science and Technology of the People’s Republic of China Foundation for SARS-nCov-02 Research (grant number 2020YFC0844500); and the Bill & Melinda Gates Foundation (award number INV-015694).

***Potential conflicts of interest.*** D. L. has a patent pending for antimicrobial infection pharmaceutical composition and its application. All other authors report no potential conflicts of interest. All authors have submitted the ICMJE Form for Disclosure of Potential Conflicts of Interest. Conflicts that the editors consider relevant to the content of the manuscript have been disclosed.
